# Putative pathophysiological mechanisms in recurrent hemicrania from aortic dissection: a case report

**DOI:** 10.1186/s13104-015-1223-8

**Published:** 2015-06-17

**Authors:** Joseph Kamtchum Tatuene, Sophie Excoffier, Jean-Paul Vallee, Andreas Kleinschmidt

**Affiliations:** Neurology Division, Department of Clinical Neurosciences, Geneva University Hospital, 4 Gabrielle-Perret-Gentil Street, 1211 Geneva 14, Switzerland; Emergency Division, Department of Community Medicine, Primary Care and Emergency Medicine, Geneva University Hospital, Geneva, Switzerland; Radiology Division, Department of Imaging and Medical Information Sciences, Geneva University Hospital, Geneva, Switzerland

**Keywords:** Headache, Hemicrania, Etiology, Pathophysiology, Aortic dissection

## Abstract

**Background:**

Transient or permanent neurological symptoms occur in 17–40% of patients with aortic dissection. They can distract from or even mask the underlying life-threatening condition.

**Case presentation:**

We present the case of a young Caucasian man who consulted for recurrent episodes of stereotyped right-sided sudden-onset severe headache. Upon questioning, he also reported a dull chest pain. Clinical examination and brain magnetic resonance imaging were unremarkable. The concomitant presence of chest pain made us consider aortic dissection. Contrast-enhanced cervico-thoraco-abdominal computerized tomography revealed type A aortic dissection. The patient underwent surgical replacement of the ascending aorta and reported no further episode of headache thereafter. Differential diagnosis of headache in this case includes paroxysmal hemicrania, cluster headache, migraine, trigeminal neuralgia and short lasting unilateral neuralgiform headache with conjunctival injection and tearing. Failure to match diagnostic criteria for any of these primary headache disorders and the resolution of pain episodes following surgery led us to postulate that these new-onset hemicrania episodes were symptomatic of aortic dissection. We hypothesize that aortic wall ischemia could have activated the trigeminovascular system and thereby caused hemicranial pain. Such an effect might be mediated by two different pathways that can be referred to as anatomical and humoral. The humoral hypothesis would posit that ischemia results in synthesis of pro-inflammatory mediators released from the aortic wall into the blood stream, such that they reach the central nervous system and directly stimulate specific receptors. The anatomical hypothesis would imply that pain signals generated by nociceptors in the aortic wall are transferred to the trigeminal ganglion via the cardiac plexus, the first cervical ganglion and the internal carotid nerve such that pain perception is referred to related cranio-cervical dermatomes.

**Conclusion:**

In cases of isolated headache that does not match key diagnostic criteria for a primary headache entity; a thorough review of systems should be performed to look for symptoms that may indicate symptomatic headache from potentially life-threatening conditions. Neurologists should consider aortic dissection in patients presenting with acute headache and chest pain. Further clinical or experimental studies are required to refute or validate the pathophysiological hypothesis discussed here.

## Background

Aortic dissection is defined as the separation of layers within the aortic wall. Typically, this diagnosis has to be considered in any patient presenting with acute tearing thoracic or abdominal pain associated with cardiovascular symptoms. Clinical manifestations are diverse, however, making the diagnosis difficult and requiring a high index of suspicion [1]. An estimated 28% of acute aortic dissections are missed on initial evaluation [2]. Although transient or permanent neurological symptoms are considered atypical manifestations, they occur in 17–40% of patients with aortic dissection and can mask the underlying life-threatening condition [3]. Previous reports have described cases where headache was the main presenting symptom of aortic dissection but pathophysiological mechanisms mediating this unusual manifestation have remained elusive [4–9]. We present the case of a young adult male who suffered recurrent, stereotyped unilateral sudden-onset severe headache as the major manifestation of a type A aortic dissection. We then discuss possible pathophysiological mechanisms that might underlie the pain, with reference to the literature.

## Case presentation

A 44-year-old non-smoking athletic Caucasian man with unremarkable medical history presented in the emergency room with unilateral right pressure-like headache. The pain had a rapid onset over 30 s and was rated 10/10 on a visual analogue scale. It was accompanied by nausea and right hemifacial hypoesthesia but not by vomiting, phonophobia, photophobia, tearing, rhinorrhea, nasal congestion, eye redness, visual impairment or motor deficit. Pain subsided within about 1 h after initiating analgesic treatment. The patient had suffered two similar headache episodes 3 and 6 days ago, rated 8/10 with spontaneous remission after 30 min. Right after the first episode, he felt a constant dull retrosternal pain rated 2/10, radiating to the back and aggravated by deep inspiration. He noticed that the subsequent episodes of hemicrania were preceded by exacerbation of the thoracic pain with radiation to the right side of the neck. A few hours after the second episode and for about 30 min, he saw flashing lights move slowly toward the midline in the right visual field, without fortification spectra and without ensuing headache. He denied any family history of migraine.

**Figure 1:**
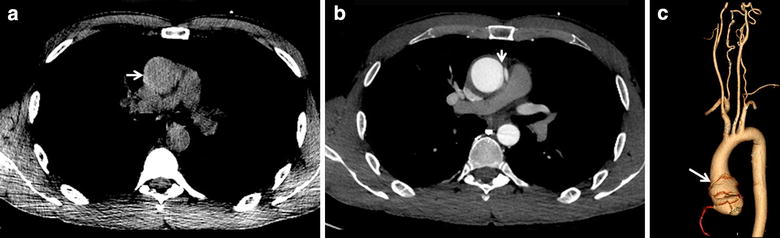
Summary of thoracic computerized tomography findings. **a** Plain axial slices demonstrating a hyperintense crescent on the right margin of the aortic lumen indicating intramural hematoma (*white arrow*). **b** Contrast enhanced axial slices demonstrating the intimal flap (*white arrow*) that underlies the pathognomonic “double lumen sign”. **c** Volume rendered reconstructed image showing the margins of the Stanford type A aortic dissection (*white arrow*). The right coronary artery and the major thoracic and cervical arteries are not involved.

Upon admission, the patient was in good general health with normal and symmetrical values of blood pressure on both arms and bradycardia (54 beats per minute). Neurological examination was unremarkable as was cardiovascular examination, including careful search for murmurs from large neck, thoracic and abdominal vessels. An electrocardiogram showed a normal sinus rhythm without signs of myocardial ischemia or infarction. Brain magnetic resonance imaging did not disclose any abnormality that could be related to the headache episodes. The concomitant presence of dull chest pain paved the way for suspecting aortic dissection. Contrast-enhanced cervico-thoraco-abdominal computerized tomography revealed type A aortic dissection sparing coronary arteries and supra-aortic vessels (Figure 1). Heart ultrasound showed an intact aortic valve. Testing for Marfan syndrome was negative. The patient underwent surgical replacement of the ascending aorta. No further episode of headache was reported after the intervention.

## Discussion

This patient suffered recurrent episodes of paroxysmal unilateral headache with unremarkable clinical examination. The headache episodes could have been superficially misclassified for instance as paroxysmal hemicrania and one could even wonder if this excruciating pain could have been the cause of the aortic dissection (sudden increase in blood pressure due to pain) rather than its consequence. Paroxysmal hemicrania is a rare primary unilateral, short-lasting (2–45 min), severe and recurrent headache with female preponderance. It is marked by ipsilateral autonomic features and exquisite responsiveness to indomethacin [10, 11]. As opposed to the original syndrome of chronic paroxysmal hemicrania described by Sjaastad [12], the term episodic paroxysmal hemicrania has been used when there was total remission between episodes [13]. Though headache episodes in our patient shared some features with primary episodic paroxysmal hemicrania, the clinical syndrome did not match due to lack of autonomic features, low frequency and longer duration of attacks and no relation with head movements (usually considered as a trigger). Other primary headache types with clinical features that overlap those of paroxysmal hemicrania to some extent, could be considered in this patient, especially cluster headache and migraine or, less likely, trigeminal neuralgia and short-lasting unilateral neuralgiform headache with conjunctival injection and tearing [11]. However, our patient did not fulfil diagnostic criteria for any of these entities. It is worth noting that the single episode of unilateral flashing lights in this case could easily have been misinterpreted as an isolated visual aura in atypical migraine.

Failure to match diagnostic criteria for any primary headache disorder led us to hypothesize that the new-onset hemicrania in this case could be symptomatic of aortic dissection. While different types of headache have been previously reported as a presenting symptom of aortic dissection [4–9], we did not find any report of recurrent sudden-onset hemicrania as a sign of aortic dissection, especially in the absence of cervical artery involvement that might explain direct lesions of surrounding trigeminal nerve fibres. To explain how aortic dissection can induce headache, some authors have alluded to distension of neck vessels or ischemia of the pericarotid plexus as potentially playing a role [7]. Conversely, we would like to suggest that headache in pure aortic dissection might originate from ischemia of the aortic wall itself.

Arterial walls are concomitantly nourished by *vasa vasorum* as well as diffusion from the lumen of the vessel. Diffusion is more important in small vessels that accordingly sometimes do not even have *vasa vasorum*. In larger vessels with a thicker wall, however, diffusion alone cannot ensure adequate supply of oxygen and nutrients to the outermost layers of the arterial wall, which makes *vasa vasorum* indispensable [14, 15]. One can imagine at least four processes by which dissection can lead to arterial wall ischemia: (1) interruption of blood flow in *vasa vasorum* due to thrombosis of the false lumen or stenosis of the true one, (2) thrombosis of *vasa vasorum* itself, (3) compression of the *vasa vasorum* by hematoma expansion in the false lumen and finally, (4) stress- and ischemia-induced reflex vasoconstriction of *vasa vasorum* due to sympathetic activation. Ischemia of the aortic wall engages different processes that might result in pathological activation of the trigeminovascular system and occurrence of cranial pain. We propose that this might happen through at least two different pathways that could be referred to as anatomical (anastomotic) and humoral.

The humoral pathway requires that inflammatory mediators formed in the aortic wall undergoing ischemia are released into the blood stream and reach the central nervous system where they can directly stimulate specific receptors. Serotonin is considered the most important of these inflammatory mediators and acts on 5 hydroxy-tryptamine 1D receptors on trigeminal perivascular projections or within trigeminal nucleus caudalis [16, 17]. Several other pain-related mediators have been described notably prostaglandins, bradykinin, adenosine triphosphate, histamine, nitric oxide, potassium and hydrogen ions. Some of them may also modulate endogenous pain-control pathways [18].

The anastomotic pathway implies that pain signals generated by nociceptors of the aortic wall are transferred to the trigeminal ganglion via the cardiac plexus. Activation of these local nociceptors is chemical, involving the aforementioned inflammatory mediators and probably also mechanical from abnormal aortic distension. The subsequent stepwise signal transfer in the anastomotic pathway relies on two anatomical peculiarities. First, small nerve fibre branches that form the cardiac plexus originate from cervical sympathetic ganglia rather than the thoracic ones [19]. Second, there is a direct connection between the first cervical ganglion and the trigeminal ganglion through the internal carotid nerve. Moreover, connections between the first cervical ganglion and the pterygopalatine and ciliary ganglia have also been described [20]. Thus, pain signals generated by nociceptors in the aortic wall undergoing ischemia could be transmitted to the trigeminal ganglion and then to the trigeminal nucleus caudalis in the lower brainstem and upper cervical cord. This information would be further relayed to the contralateral ventral posteromedial thalamic nuclei via the trigeminothalamic tract and finally to somatosensory cortices [21]. Since different levels of representation also integrate various signals from cephalic trigeminal dermatomes, one could imagine that such convergence of pain signals could induce a situation where pain arising from damage to the aorta is felt on the face as is the case in other situations of projected or referred pain [22].

Headache does not always occur in aortic dissection and when it does, it does not usually present as episodic paroxysmal unilateral headache. In most cases of aortic dissection, pain is referred to the thoracic wall and hence felt in the chest rather than in the head. Two hypotheses could be suggested to provide a comprehensive understanding of these clinical facts. First, one could imagine that patients who present with headache from aortic dissection may have anatomical (more connections) and functional (more permissive) variants of the anastomotic circuits between the cardiac plexus and the trigeminal ganglion. Second, patients with aortic dissection whose headache mimics migraine or other short-lived trigeminal autonomic syndromes may have a genetic background modulating the probability of their trigeminovascular system being activated by circulatory inflammatory mediators.

With currently available data, it remains impossible to determine which of the two mechanisms we propose might play the greater role and why patients sometimes have unilateral rather than bilateral pain.

## Conclusion

In cases of isolated headache that does not match key diagnostic criteria for a primary headache entity; a thorough review of systems should be performed to look for subtle or atypical symptoms that may indicate symptomatic headache from potentially life-threatening conditions. In particular, neurologists should consider aortic dissection in patients presenting with acute headache and even mild chest pain. The relation between headache, especially hemicrania, and intra-thoracic diseases should not be considered as anecdotal: it could be well explained by pathological activation of the trigeminovascular system through anastomotic and/or humoral pathways. Nonetheless, the putative pathophysiological mechanisms discussed here still require validation in future clinical and experimental studies.

### **Consent**

Written informed consent was obtained from the patient for publication of this Case report and any accompanying images. A copy of the written consent is available for review by the Editor-in-Chief of this journal.
